# Alcohol Sensing Behavior and Impedance Spectroscopy Characterization of g-C_3_N_4_ Nanosheets

**DOI:** 10.3390/nano16030213

**Published:** 2026-02-06

**Authors:** Cong Doan Bui, Svetlana Nalimova, Valery Kondratev, Zamir Shomakhov, Svetlana Kirillova, Alexander Maximov, Vyacheslav Moshnikov

**Affiliations:** 1 Department of Micro- and Nanoelectronics, Saint Petersburg Electrotechnical University “LETI”, Professora Popova St. 5, Saint Petersburg 197022, Russia; sskarpova@list.ru (S.N.); aimaximov@mail.ru (A.M.); vamoshnikov@mail.ru (V.M.); 2 Moscow Center for Advanced Studies, Kulakova St. 20, Moscow 123592, Russia; kvm@mail.ru; 3 Center for Nanotechnologies, Alferov University, Khlopina St. 8/3, Saint Petersburg 194021, Russia; 4 Institute of Artificial Intelligence and Digital Technologies, Kabardino-Balkarian State University, Chernyshevsky St. 173, Nalchik 360004, Russia; shozamir@yandex.ru; 5 Institute of Informatics and Regional Management Problems, Kabardino-Balkarian Scientific Center RAS, Balkarova St. 2, Dolinsk 360002, Russia; 6 Department of Physical Chemistry, Saint Petersburg Electrotechnical University “LETI”, Professora Popova 5, Saint Petersburg 197022, Russia; refractory-sveta@mail.ru

**Keywords:** g-C_3_N_4_, gas sensor, alcohol detection, impedance spectroscopy, charge transfer resistance, constant phase element

## Abstract

Two-dimensional graphitic carbon nitride 2D g-C_3_N_4_ has the potential for gas sensing as a metal-free semiconductor with a layered structure, high surface area, and tunability of electronic properties. In this context, 2D g-C_3_N_4_ nanosheets were prepared by the thermal polycondensation of urea followed by ultrasonic exfoliation. X-ray diffraction revealed diffraction peaks corresponding to the (110) and (002) crystallographic planes of g-C_3_N_4_. Scanning electron microscopy showed a nanosheet structure with a 10-nm crystallite size, while energy-dispersive X-ray spectroscopy demonstrated a uniform distribution of carbon and nitrogen. Ultraviolet–visible absorption spectroscopy revealed a band gap of 2.8 eV. Gas sensing measurements exhibited an increase in response to isopropanol and ethanol as the operating temperature and gas concentration increased. Impedance spectroscopy provided additional insight into the sensing mechanism. Observed depressed semicircles in Nyquist plots were fitted with a charge transfer resistance R_ct_ in parallel with a constant phase element model. The charge transfer resistance R_ct_ fell systematically with isopropanol exposure, confirming the crucial role of adsorption-induced electron transfer in the gas sensing response.

## 1. Introduction

Maintaining air quality is crucial for human health. It involves ensuring that the atmosphere is free from harmful gases and pollutants. However, the increase in population, socioeconomic development, industrialization and exploitation of natural resources are likely to lead to a deterioration in air quality. Detection of volatile organic compounds (VOCs), such as isopropanol (IPA) or ethanol, assumes major significance. Regarding industrial application, IPA and ethanol are widely used as solvents and fuels. Their properties of volatility and flammability must be continuously monitored to ensure industrial safety [1]. Moreover, breath analysis has recently been found to be a new non-invasive approach to biomarker detection in medicine [2]. Specific amounts of IPA or alcohol derivatives present in human breath are identified as diagnostic markers of pulmonary ailments or diabetes [3]. Finally, the control of the amount of ethanol helps to determine the level of fermentation, which can be used to estimate the freshness of the relevant commodities [4].

Over the past thirty years, a considerable number of materials have been explored for use in chemosensing applications. Some of them, such as semiconductor metal oxides (SMOs), including WO_3_ [5,6], ZnO [7,8], SnO_2_ [9,10], and TiO_2_ [11,12], have received a lot of attention due to their potential for creating gas sensors. These materials are used because of their favorable electronic properties, thermal and chemical stability, ease of fabrication, and cost-effectiveness. Nevertheless, sensors fabricated using traditional SMOs usually have poor selectivity and thermal stability, and often require high operating temperatures [13]. The high thermal requirements restrict their application in portable alcohol detectors due to excessive power consumption and safety concerns regarding flammable vapors. To overcome these limitations, researchers have focused on new nanostructured materials.

Graphitic carbon nitride (g-C_3_N_4_) is a low-cost, abundant and easily scalable semiconductor nanomaterial. It has a graphite-like structure consisting of layers of van der Waals bonded sp^2^ hybridized nitrogen and carbon atoms. The ideal form of the material consists of aromatic sheets made up of heptazine and triazine units held together by weak van der Waal forces [14,15,16,17]. g-C_3_N_4_ has good electronic properties, excellent thermal and chemical stability, and a band gap of about 2.7 eV [18,19]. For nanosheets, the band gap is slightly larger, ranging from 2.78 to 2.82 eV compared to 2.67 to 2.72 eV in bulk material. This difference is due to the quantum confinement effects [20,21].

The two-dimensional (2D) nature of g-C_3_N_4_ exhibits extremely fascinating properties for sensing applications. Its 2D layered structure provides a rich array of active surface sites, which contribute to its high specific surface area. This increased surface area provides more interaction sites for gas molecules, making g-C_3_N_4_ more effective at gas sensing [22,23,24].

Suitable physicochemical properties and tunable electronic structure have led to the future use of 2D g-C_3_N_4_ in gas-sensing devices. Although g-C_3_N_4_ shows enormous potential for use in chemosensing applications, little research has been conducted on pure g-C_3_N_4_ and composite-based gas sensors [25]. Specifically, the intrinsic charge transfer mechanism of alcohol molecules on pure 2D g-C_3_N_4_ nanosheets remains underexplored compared to metal-oxide composites. While g-C_3_N_4_ has been extensively investigated for other mechanisms such as photoelectrochemical and fluorescence-based sensing, it is difficult to find a comprehensive review of its chemiresistive gas sensing properties [26,27].

Electrochemical impedance spectroscopy (EIS) is a highly sensitive method that is superior to traditional approaches. EIS can provide detailed information regarding the mechanism and kinetics of a process as well as unique insights into the detection process [28,29].

However, despite its strengths in analyzing multidimensional and dynamic electronic processes, the direct application of EIS to a detailed examination of gas sensing properties in g-C_3_N_4_ sensors is currently not available. Therefore, using EIS is essential for a better understanding of charge transport dynamics in g-C_3_N_4_ chemiresistive gas sensors.

The main goal of this work was to synthesize 2D g-C_3_N_4_ nanosheets and investigate their structure, morphology, composition and optical properties as well as study their gas sensing behavior towards VOCs. The major contribution and novelty of our work is the detailed mechanistic insight provided by EIS analysis, which allows us to fully describe the alcohol sensing mechanism using a correct equivalent circuit model. We used a parallel (R_ct_||CPE) circuit to accurately describe the non-ideal capacitance response commonly observed in nanostructured materials due to the effects of surface roughness or heterogeneity. This systematic approach has provided a comprehensive explanation of the charge transfer process involved in the interaction between gas molecules and the 2D g-C_3_N_4_ sensing layer.

## 2. Materials and Methods

### 2.1. Synthesis of 2D g-C_3_N_4_ and Gas Sensor Fabrication

A total of 10 g of urea was placed in a sealed crucible and subjected to thermal polycondensation in a muffle furnace at 550 °C for 4 h. The yellow product was then washed with a solution of HNO_3_, IPA, and distilled water until the pH reached 7. The residue was then air-dried at 80 °C for 12 h to yield a pure bulk g-C_3_N_4_ powder.

g-C_3_N_4_ powder was dispersed in water using ultrasonic treatment for 1 h to achieve partial exfoliation and obtain a stable colloidal suspension. This suspension was applied evenly to a ceramic sensor platform using five cycles of spin coating (2000 rpm, 15 s). Thermal treatment at 450 °C for 30 min ensured good adhesion and structural integrity, forming a sensitive element with high mechanical strength.

### 2.2. Characterization

The crystal structure was analyzed by X-ray diffraction (XRD) using a DRON-8N diffractometer (Bourevestnik JSC, Saint Petersburg, Russia) with Cu Kα radiation (λ = 1.54056 Å) in the angular range 2θ from 10° to 70°. The microstructure of g-C_3_N_4_ was studied by scanning electron microscopy (SEM), and its elemental composition was examined by energy-dispersive X-ray spectroscopy (EDX) using a Zeiss Supra 25 (Zeiss, Oberkochen, Germany) scanning electron microscope. The absorption spectra were measured on a PE-5400UV spectrophotometer (ECROS Group, Saint Petersburg, Russia).

### 2.3. Measurement of Sensing Properties

**Figure 1:**
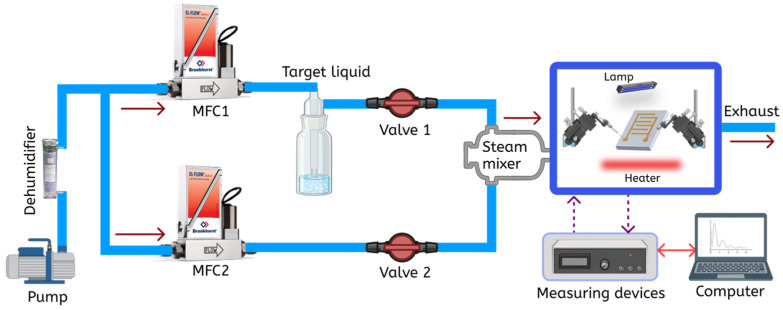
System for generating vapor flows and measuring gas sensing properties.

A special laboratory setup, shown in Figure 1, was used to investigate the response of the synthesized gas-sensitive layers to IPA, ethanol, and acetone. The system produces vapor streams and analyzes gas-sensing properties by detecting changes in electrical resistance or impedance when exposed to specific vapors. An air pump delivers dry air into two channels, which are regulated by mass flow controllers (MFCs). One channel delivers the target liquid vapor (e.g., IPA, ethanol, acetone) from a bubbler while the other channel supplies dehumidified ambient air. These vapors are mixed and fed into a chamber where a sample is placed. The final concentration (C) in the measurement chamber is determined based on the saturation vapor pressure of the liquid at room temperature and the ratio of flow rates, according to Equation (1):

(1) $$C=\frac{P_{gas}\times F_{gas}}{P_{atm}\times (F_{gas}+F_{air})}$$

where P_gas_ is the saturated vapor pressure of the bubbled liquid, P_atm_ is the atmospheric pressure, F_gas_ is the air flow rate through the bubbler, and F_air_ is the diluent air flow rate. By adjusting the ratio of F_gas_ to the total flow, we can accurately establish target concentrations for our experiments.

Instrumentation tools include a sample chamber, power supply modules, and control elements. The current was measured using a Keithley 6485 Picoammeter (Keithley, Cleveland, OH, USA). Impedance spectra were measured in the frequency range from 1000 Hz to 500 kHz using the Z-500P Impedance Analyzer (Elins, Moscow, Russia). The amplitude of the alternating signal was 255 mV and the bias voltage was 1 V. An Arduino controller was used to control system parameters such as valves and environmental conditions. The measurements were carried out at 150, 200 and 250 °C, followed by purging of the sample using dried air for 10 min.

The sensing parameters are described as follows [30,31]:

1. Response (R_a_/R_g_): The response of n-type semiconductor gas sensors, such as the 2D g-C_3_N_4_ sensor, to reducing gases like IPA or ethanol is given by comparing their electrical resistances in dry air (R_a_) and in the presence of the target gas (R_g_).
2. Response Time (τ_res_): This is the time it takes for the resistance of the sensor to reach 90% of its total resistance change after exposure to the test gas.
3. Recovery Time (τ_rec_): This parameter is the time taken for the resistance of a sample to recover 90% of its total resistance change after the target gas has been removed and the chamber has been flushed with air.

## 3. Results and Discussion

### 3.1. Morphology Analysis

**Figure 2:**
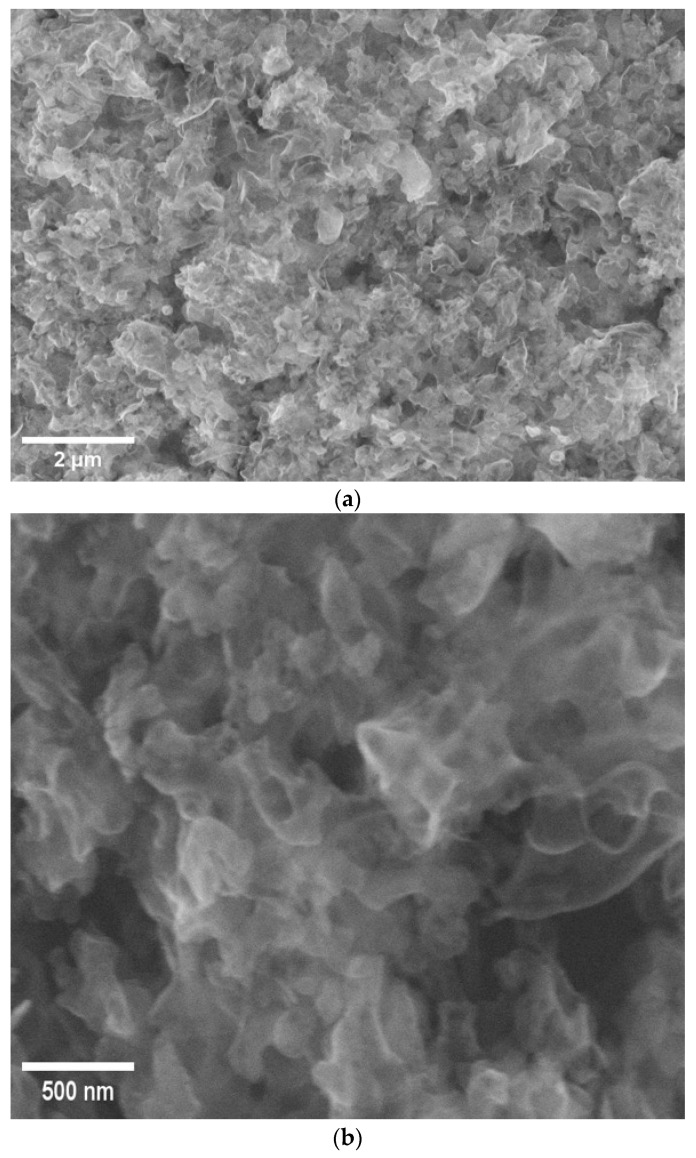
SEM images of 2D g-C_3_N_4_ with different magnifications: (**a**) 25,000×; (**b**) 100,000×.

The morphology of the g-C_3_N_4_ sample at a magnification of 25,000× is presented in Figure 2a. The material has a conventional layered structure, which is characterized by loosely superimposed and irregularly overlapping crumpled nanosheets.

The nanosheets have lateral sizes ranging around 200–500 nm (Figure 2b). The wrinkled surface and interlayer pores create a porous structure, significantly increasing the available surface area. This morphological feature contributes to improved gas sensing performance by facilitating charge transfer and enhancing the diffusion of gas molecules.

In general, the SEM images show that the produced g-C_3_N_4_ consists of disordered few-layer nanosheets with high surface roughness.

### 3.2. XRD Analysis

**Figure 3:**
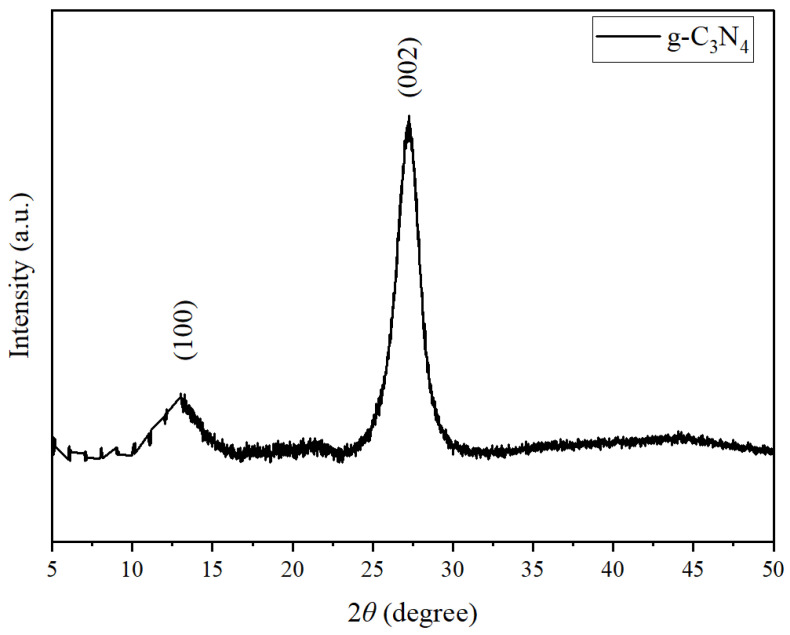
XRD pattern of 2D g-C_3_N_4._

The XRD pattern of the as-prepared g-C_3_N_4_ nanosheets is shown in Figure 3. There were two characteristic diffraction peaks observed at 2θ ≈ 13.1° and 2θ ≈ 27.3°. These peaks were assigned to the (002) and (100) crystallographic planes, respectively.

The (100) peak at 13.1° is due to the in-plane structural patterns of tri-s-triazine packing units, which correspond to the periodicity of the heptazine backbone of the g-C_3_N_4_ fragments. The (002) peak at 27.3° is due to the interlayer stacking of conjugated aromatic systems, similar to the (002) reflectance of graphite with an interlayer distance of about 0.326 nm, as calculated using Bragg’s law [32,33,34].

Interestingly, the (002) peak of the sample was broader and less sharp than the corresponding peak in bulk g-C_3_N_4_ [35,36]. This suggests a reduction in the stacking order along the c-axis due to partial exfoliation. The crystallite size was calculated using the Scherrer Equation (2):

(2) $$D=\frac{K\lambda }{\beta \cos\theta },$$

where K = 0.9, λ = 0.15406 nm (Cu Kα radiation), β = 0.8° in radians, and θ = 13.65. This was determined to be approximately 10.2 nm. The relatively small crystallite size indicates that the material consists of few-layer nanosheets rather than bulk structures. This was confirmed by scanning electron microscopy analysis of the wrinkled and loosely packed layers.

This structural aspect is beneficial for gas sensing applications The thinner layer and larger interlayer distance facilitate the easier migration of charge carriers, expose more active sites, and improve the diffusion of reactant molecules.

### 3.3. EDX Analysis

**Figure 4:**
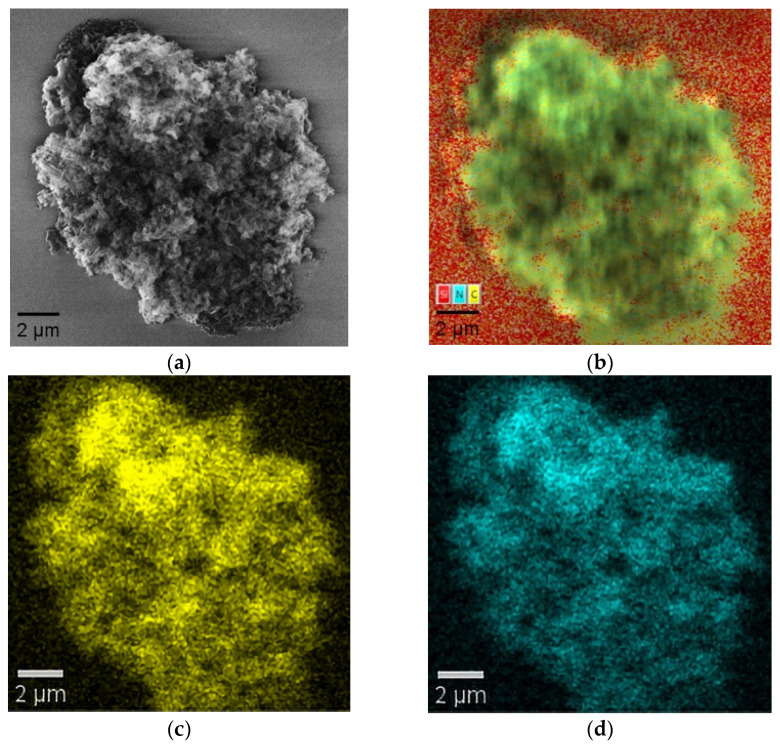
SEM image of 2D g-C_3_N_4_ (**a**); EDX elemental mapping (**b**); C Kα elemental map (**c**); and N Kα elemental map (**d**).

The SEM image, elemental mapping, and EDX distribution of carbon (C) and nitrogen (N) in the as-synthesized g-C_3_N_4_ nanosheets are shown in Figure 4.

EDX mapping (Figure 4b) shows a uniform distribution of C and N elements on the surface of the particle. The signal corresponding to silicon is related to the composition of the substrate used in this experiment. C Kα and N Kα elemental mapping (Figure 4c,d) also revealed a uniform distribution of carbon and nitrogen. The absence of any major foreign elements indicates that the material is highly pure.

The uniform distribution of elements in the composition ensures that the process of thermal exfoliation and polymerization results in a chemically homogeneous 2D material. This compositional homogeneity is important for gas sensing applications as it provides reproducible performance and uniform electronic properties.

### 3.4. Optical Absorption and Band Gap Estimation

**Figure 5:**
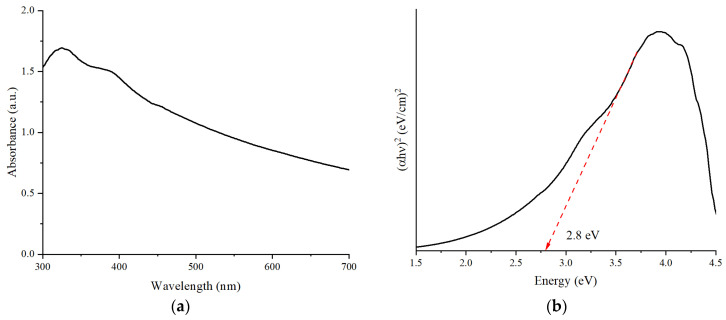
(**a**) UV–Vis absorption spectrum. (**b**) Tauc plot versus photon energy of 2D g-C_3_N_4_.

Figure 5 shows the UV–Vis absorption spectrum of the as-synthesized 2D g-C_3_N_4_. It had a high absorption edge at 350 nm, indicating that the material predominantly absorbs ultraviolet light. The absorption gradually decreased towards the visible range (450–700 nm), suggesting that g-C_3_N_4_ has a low but not insignificant absorption of visible light. This phenomenon is due to electronic transitions within the conjugated heptazine network.

To determine the optical band gap, a Tauc plot was created by plotting (αhν)^2^ against photon energy (hν), as shown in Figure 5b. From the linear part of the plot, the optical band gap was estimated to be 2.8 eV by extrapolating it to the energy axis. This value corresponds with the findings of other researchers [37,38,39,40].

### 3.5. Gas-Sensing Performance

**Figure 6:**
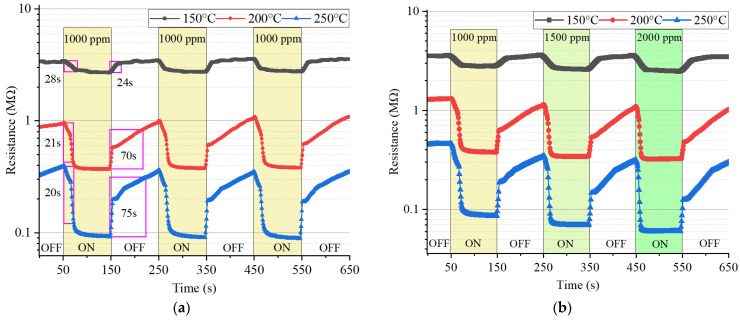
(**a**) Dynamic response–recovery curves of the 2D g-C_3_N_4_ gas sensor towards 1000 ppm IPA. (**b**) Response curves of the sensor towards various IPA concentrations at the same three operating temperatures.

The dynamic gas-sensing characteristics of the 2D g-C_3_N_4_-based sensor to IPA under different operating conditions are shown in Figure 6.

Figure 6a shows the response–recovery curves of the sensor to 1000 ppm IPA at different operating temperatures (150 °C, 200 °C, 250 °C). The response of the sensor, R_a_/R_g_, increases significantly with temperature. The highest response was at 250 °C, with a value of 4.5. At 150 °C, the response was only 1.3. Higher temperature changes the form of chemisorbed oxygen species [41,42] and surface redox reactions with IPA molecules, leading to a larger resistance variation. The rapid recovery at 250 °C also suggests good desorption and reversibility of the sensor. Multiple response cycles to three consecutive exposures support good stability and reproducibility of the sensor. Response and recovery times of 20 and 75 s (indicated by pink rectangles) were observed for the g-C_3_N_4_ sensor at the optimal operating temperature of 250 °C for the IPA target. Figure 6 shows that the response curve can be divided into two stages: a linear part at the initial stage, and then an exponential part, related to the intergrain boundaries. The recovery curve had the same stages: a sharp decrease associated with the formation of depleted regions at the intergrain boundaries, followed by a linear part.

Figure 6b indicates the response to different concentrations of IPA (1000, 1500, 2000 ppm) at the same temperatures. The response values were calculated based on independent resistance measurements taken at various temperatures and concentrations of the target gas. Response rose nearly with concentration, exhibiting a surface absorption-controlled sensing mechanism. For 250 °C, the response was more than 8 for 2000 ppm, which supports high sensitivity.

These findings show that the optimal working temperature for 2D g-C_3_N_4_ to detect IPA is 250 °C with large response amplitude, short response/recovery times 20 s/75 s, and reproducibility. The quasi-linear concentration–response relationship makes it beneficial for quantitative gas sensing.

The response value in adsorption-type sensors is influenced not only by the gas molecules that interact with the sensor surface, but also by oxygen and water molecules that are involved in the detection process. During detection, water is formed as a result of the reaction between the reducing gas and oxygen. Gas adsorption and desorption processes are influenced by changes in the partial pressures of water and oxygen in the atmosphere. These fluctuations can lead to differences in the results when measurements are taken on different days, but for most applications, they are not significant enough to affect the interpretation of the results. Therefore, there are slight differences in the response values calculated based on the data presented in Figure 6a,b.

A comparison of the responses of the 2D g-C_3_N_4_ gas sensor to various VOCs with different concentrations at different operating temperatures is shown in Table 1. It was found that there was no response to acetone at 150 °C. In other cases, the response to acetone was significantly lower than to ethanol and IPA. At operating temperatures of 150 °C and 200 °C, the responses to alcohol vapors were close, and at 250 °C, the response to isopropyl alcohol slightly exceeded the response to ethanol. Thus, among the three studied volatile organic compounds, the developed sensor makes it possible to detect alcohols, which may be due to their chemical nature and mechanism of interaction with the semiconductor surface.

The sensor characteristics obtained in this work are comparable to those of other researchers who have studied g-C_3_N_4_ composites with different metal oxides. Since operating temperature is a significant factor in determining the performance and energy efficiency of sensors, studies conducted at operating temperatures close to 250 °C were considered. We compared the characteristics of ethanol detection, as there is no information on the detection of IPA. In Ref. [43], a SnO_2_-g-C_3_N_4_ composite with 7 vol. % g-C_3_N_4_ was tested for its response to 500 ppm ethanol at 260 °C, and the R_a_/R_g_ ratio was approximately 10. It was shown in [44] that the addition of g-C_3_N_4_ to a CuO/ZnO heterojunction increased the response to 500 ppm ethanol up to 14 at an operating temperature of 240 °C. In Ref. [45], α-Fe_2_O_3_-g-C_3_N_4_ nanocomposite (g-C_3_N_4_ content—40 wt. %) at an operating temperature of 260 °C showed a response to 100 ppm of ethanol equal to 3.5. At the same conditions, the response of pure g-C_3_N_4_ was approximately 1. In one of the works [46], the possibility of detecting ethanol at lower temperatures was shown. A nanocomposite g-C_3_N_4_-ZnO-Zn_2_SnO_4_ (g-C_3_N_4_ content—5 wt. %) was developed for this purpose. At 30 °C, this sensor showed a response to 100 ppm of ethanol equal to 14.6. It should be noted that the relative humidity in this experiment was 40%, and as it decreased and increased, the response decreased. These results are summarized in Table 2.

The response and recovery kinetics in this study were quite fast compared to those for other reported g-C_3_N_4_ systems. For instance, response times to NO_2_ of up to 142 s have been reported for ZnO/g-C_3_N_4_-based gas sensors, and recovery times for SnS_2_/g-C_3_N_4_ composites are about 166 s [47]. The quick response of the current material can be explained by the thin exfoliated nanosheets of the 2D g-C_3_N_4_ sensor.

The values of the sensing response reported in this work are lower than some reported values in the literature because those studies used g-C_3_N_4_ as an additive in metal-oxide composites, which improves the sensing response through heterojunction effects, while this work focused on the properties of pure 2D g-C_3_N_4_. However, it should be noted that the response of 4.5 reported in this work is still higher than the reported response of approximately 1 reported in previous comparative works for pure g-C_3_N_4_. The use of pure 2D g-C_3_N_4_ has important practical advantages over traditional metal oxides including lower manufacturing costs, higher structural stability, and a minimized risk of surface poisoning.

### 3.6. EIS Analysis

**Figure 7:**
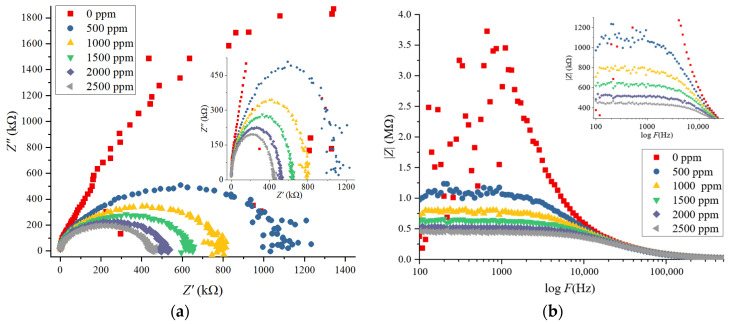
(**a**) Nyquist plots of the 2D g-C_3_N_4_ gas sensor at different IPA concentrations (0–2500 ppm). (**b**) Bode magnitude plots of the 2D g-C_3_N_4_ gas sensor at different IPA concentrations (0–2500 ppm).

The EIS responses of the 2D g-C_3_N_4_ sensor at varied IPA concentrations (0–2500 ppm) at 250 °C are represented in Figure 7. The EIS responses were measured using a 1 V DC signal, and the impedance response was explored through the Nyquist (Z″ vs. Z′), and Bode magnitude (|Z| vs. frequency) plots.

The Nyquist plots in Figure 7a show a series of semicircular arcs followed by a straight line at low frequencies, characteristic of charge transfer resistance and diffusion-controlled processes, respectively. The semicircular arc diameter is reduced when the IPA concentration is higher, consistent with the decrease in R_ct_.

• Zero ppm IPA showed a comparatively high charge transfer resistance (1.6 MΩ), indicating that electron flow is not possible in the absence of gas molecules.
• At 1000 ppm IPA, the arc diameter reduced to about 0.8 MΩ, representing a drastic reduction in R_ct_, caused by the absorption of IPA molecules on the surface of g-C_3_N_4_, making electron transfer easier.
• At 2500 ppm IPA, the value of R_ct_ decreased to about 300 kΩ, proving that there is an increase in the injection of electrons into the material at a higher concentration of IPA, resulting in lower overall resistance.

The inset in Figure 7a indicates the zoomed-in impedance spectra, where arcs become more pronounced with increasing concentration of IPA, indicating an ideal fitting to the equivalent circuit and proving the surface-controlled nature of the sensing process.

The Bode magnitude plots in Figure 7b show the frequency dependence of impedance magnitude with varying IPA concentrations. With increasing IPA concentration, impedance decreases throughout the entire frequency range, verifying that adsorption of IPA improves electron conduction.

• At 0 ppm IPA, there was a high impedance magnitude (3.5 MΩ at 1 kHz), which indicates a low charge carrier concentration and a high resistance of g-C_3_N_4_.
• Impedance magnitude fell to approximately 2.8 MΩ at 500 ppm IPA, indicating a moderate increase in the charge carrier concentration.
• The impedance also reduced at 1000 ppm IPA to 2.2 MΩ, exhibiting a greater response as more IPA molecules become adsorbed.
• The impedance decreased to 1.3 MΩ at 2500 ppm IPA (the inset in Figure 7b), confirming the presence of a high concentration of IPA molecules adsorbed on the surface, with more electrons returning to the conduction band of g-C_3_N_4_ and decreasing the overall resistance. This trend continues across all frequencies, particularly in the low- to mid-frequency range, where gas–solid interactions are dominant.

The impedance behavior of the 2D g-C_3_N_4_ sensor was modeled using an equivalent circuit consisting of R_ct_ in parallel with CPE [48,49,50]. Within the model, the CPE replaces an ideal double-layer capacitor to represent the non-ideal capacitive behavior of porous and heterogeneous surfaces that are characteristic of 2D nanomaterials [51,52].

The impedance of the CPE is given by Equation (3):

(3) $$Z_{CPE}=\frac{1}{{Q\times (j2\pi f)}^{\alpha }}\;,$$

where *Q* is the CPE constant (with units Ω^−1^ × s^α^), α (0 ≤ *α* ≤ 1) is the phase factor, *f* is the frequency, and *j* is the imaginary unit. When *α* = 1, the CPE is an ideal capacitor with capacitance *C* = *Q*. When *α* = 0, it is an ideal resistor with resistance *R* = 1/*Q*. For intermediate values (0 < *α* < 1), the CPE represents a non-ideal capacitive response of electrode surface roughness, porosity, and distributed relaxation time on the electrode surface.

The fitted parameters in Table 3 set a distinct trend in the impedance response of the 2D g-C_3_N_4_ sensor to IPA exposure.

The R_ct_ is systematically reduced from 11.3 × 10^5^ Ω at 500 ppm to 4.52 × 10^5^ Ω at 2500 ppm, which shows that increased IPA concentrations cause electron transfer to occur over the sensor surface. In CPE, the constant Q and phase factor α, however, remain constant over all concentrations, which indicates that the capacitive response at the interface is stable and controlled by the inherent structure of g-C_3_N_4_.

**Figure 8:**
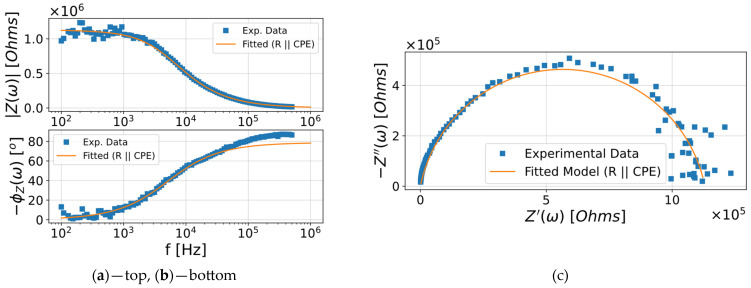
Experimental impedance spectra (blue) and equivalent circuit fitting (orange) of 2D g-C_3_N_4_ sensor exposed to 500 ppm IPA, represented as the (**a**) Bode magnitude plot, (**b**) Bode phase plot, and (**c**) Nyquist plot.

The satisfactory fit of the impedance spectra for 500 ppm IPA using the equivalent circuit model (R_ct_||CPE) suggests that there is a good agreement between the experimental data and simulated curve (Figure 8).

For the Nyquist plot (Figure 8c), the experimental results showed a depressed semicircle, which is well-accounted for by the CPE element. This indicates the non-ideal capacitive behavior of the 2D g-C_3_N_4_ surface. For the Bode magnitude plot (Figure 8a), the impedance |Z| decreased with frequency, and the phase plot (Figure 8b) showed a maximum phase angle of 80°. This is in agreement with the strong capacitive contribution. The fit closely follows these two trends, further supporting the applicability of the chosen equivalent circuit. Phase differences between the experimental and fitted values in the high-frequency range may be due to several factors including the limitations of (R_ct_||CPE) model, wiring parasitic inductance, or measurement noise at very high frequencies.

### 3.7. Sensing Mechanism

The sensing mechanism of the 2D g-C_3_N_4_ nanosheets towards alcohols such as IPA and ethanol is based on the surface-adsorbed oxygen ion model, which is characteristic of n-type semiconductor chemiresistors. This process involves several distinct stages: adsorption, surface reaction, charge transfer, and desorption.

1. Oxygen adsorption.

When the g-C_3_N_4_ sensor is exposed to ambient air, O_2_ molecules are physically and then chemically adsorbed onto the surface of the nanosheets. Due to the high electron affinity of oxygen, these molecules capture free electrons from the conduction band of the n-type g-C_3_N_4_ to form anionic oxygen species such as $O_{2}^{-}$, $O^{-}$, or $O^{2-}$, depending on the operating temperature. This extraction of electrons leads to the formation of an electron depletion layer (EDL) at the surface and an increase in the potential barrier at the grain boundaries. Consequently, the baseline resistance R_a_ is high.

2. Surface reaction and charge transfer.

Upon exposure to reducing gases like IPA or ethanol, the gas molecules diffuse into the porous network of the g-C_3_N_4_ nanosheets and adsorb onto the active sites. These molecules then undergo a redox reaction with the pre-adsorbed oxygen ions.

Reaction example (ethanol/IPA):

$$C_{2}H_{5}OH\left(ads\right)+6O^{-}\left(ads\right)\to 2CO_{2}+3H_{2}O+6e^{-}$$

As a result of this reaction, the electrons previously trapped by the oxygen species are released back into the conduction band of the g-C_3_N_4_. This charge transfer increases the carrier concentration, narrows the width of the electron depletion layer, and lowers the potential barrier at intergrain interfaces.

3. Resistance variation

Because g-C_3_N_4_ behaves as an n-type semiconductor, the injection of electrons from the target gas molecules leads to a significant decrease in electrical resistance to the R_g_ value. Our impedance spectroscopy results provide direct experimental evidence for this; the R_ct_ was found to fall systematically as the concentration of IPA increased (e.g., from 1.6 MΩ at 0 ppm to approximately 452 kΩ at 2500 ppm), verifying that adsorption-induced electron transfer is the dominant sensing pathway.

4. Desorption and recovery

When the target gas is removed and air is reintroduced, the reaction products (CO_2_ and H_2_O vapor) desorb from the sensor surface. Atmospheric oxygen once again adsorbs onto the g-C_3_N_4_ nanosheets, capturing electrons to reform the anionic species and rebuild the electron depletion layer. This process restores the potential barrier and causes the sensor resistance to return to its original high baseline value R_a_, completing the sensing cycle.

## 4. Conclusions

Structural, compositional, and optical characterizations confirmed the successful synthesis of 2D g-C_3_N_4_ with maximized gas sensing properties. SEM indicated rumpled nanosheets with a high surface area, and XRD indicated crystalline peaks with a nanoscale size of the crystallites (10 nm). EDX mapping confirmed a uniform distribution of C and N, hence chemical homogeneity and the absence of impurities. The UV–Vis spectrum showed strong absorption in the visible region and a band gap of 2.8 eV, as has been already reported, ascertaining the semiconductor nature of g-C_3_N_4_ for charge transfer reactions.

Gas-sensing performance testing showed a reproducible response to IPA, with increased sensitivity at higher temperatures and higher concentrations. Impedance analysis provided additional information. Nyquist plots revealed depressed semicircles that were were well-fitted by the (R_ct_||CPE) circuit, characterizing non-ideal capacitive behavior typical for nanostructured surfaces. A systematic reduction in R_ct_ with increasing IPA concentration suggested that electron transfer was the predominant sensing mechanism. The constant CPE values indicated that the interfacial capacitance was predominantly intrinsic to g-C_3_N_4_.

The findings are consistent with previous studies of 2D semiconductors and support the hypothesis that adsorption–desorption dynamics play a significant role in regulating R_ct_. The research suggests that g-C_3_N_4_ may be a potential material for VOC sensing due to its structural stability and efficient electron transport. In the future, its selectivity could be enhanced through surface modification and heterojunction engineering. In situ spectroscopic studies could provide deeper insights into adsorption dynamics. Future studies will also need to evaluate device integration, ambient stability, and long-term performance in order to drive g-C_3_N_4_ towards practical sensing technology.

## Figures and Tables

**Table 1:** Sensor responses of 2D g-C_3_N_4_ at different temperatures and gas concentrations.

Temperature, °C	Concentration, ppm	Response R_a_/R_g_		
		Isopropanol	Ethanol	Acetone
150	1000	1.28	1.12	1.00
	1500	1.39	1.16	1.00
	2000	1.45	1.25	1.00
200	1000	3.41	3.28	1.04
	1500	3.78	3.61	1.05
	2000	4.01	3.92	1.08
250	1000	5.25	4.54	1.39
	1500	6.56	5.52	1.54
	2000	7.58	6.23	1.66

**Table 2:** Ethanol sensing characteristics of various g-C_3_N_4_-based materials.

Sensing Material	Concentration, ppm	Operating Temperature, °C	Response, R_a_/R_g_	Reference
Pure 2D g-C_3_N_4_	1000	250	4.5	Present work
SnO_2_-g-C_3_N_4_ (7 vol. %)	500	260	~10	[43]
g-C_3_N_4_-CuO/ZnO	500	240	14	[44]
α-Fe_2_O_3_-g-C_3_N_4_ (40 wt. %)	100	260	3.5	[45]
Pure g-C_3_N_4_	100	260	~1	[45]
g-C_3_N_4_-ZnO-Zn_2_SnO_4_ (5 wt. %)	100	30	14.6	[46]

**Table 3:** Fitted equivalent circuit parameters (R_ct_, Q, α) obtained from impedance spectra of the 2D g- C_3_N_4_ sensor under exposure to IPA at different concentrations.

Concentration, ppm	R_ct_, 10^5^ Ω	Q, 10^−10^ Ω^−1^ × s^α^	α
500	11.3	1	0.875
1000	8.02	1	0.875
1500	6.47	1	0.875
2000	5.26	1	0.875
2500	4.52	1	0.875

## Data Availability

Data are contained within the article.

## Abbreviations

The following abbreviations are used in this manuscript:

2D	Two-dimensional
AC	Alternating current
CPE	Constant phase element
DC	Direct current
EDX	Energy-dispersive X-ray spectroscopy
EIS	Electrochemical impedance spectroscopy
g-C_3_N_4_	Graphitic carbon nitride
MFC	Mass flow controller
SEM	Scanning electron microscopy
SMOs	Semiconducting metal oxides
VOC	Volatile organic compound
XRD	X-ray diffraction
